# Progress Toward Measles Elimination — Eastern Mediterranean Region, 2008–2012

**Published:** 2014-06-13

**Authors:** Nadia Teleb, Emmaculate Lebo, Hinda Ahmed, Abdel Rahman Hossam, El Tayeb El Sayed, Alya Dabbagh, Peter Strebel, Paul Rota, James Alexander

**Affiliations:** 1 Vaccine Preventable Diseases and Immunization, WHO Regional Office for the Eastern Mediterranean, Cairo, Egypt; 2 Global Immunization Division, Center for Global Health, CDC; 3 Department of Immunization, Vaccines, and Biologicals, WHO, Geneva, Switzerland; 4 Division of Viral Diseases, National Center for Immunization and Respiratory Diseases, CDC

In 1997, the 22 countries in the World Health Organization (WHO) Eastern Mediterranean Region (EMR)* adopted a goal of measles elimination by 2010† (1). To achieve this goal, the WHO Regional Office for the Eastern Mediterranean Region (EMRO) developed a four-pronged strategy: 1) achieve ≥95% vaccination coverage of children with the first dose of measles-containing vaccine (MCV1) in every district of each country through routine immunization services, 2) achieve ≥95% vaccination coverage with the second dose of measles-containing vaccine (MCV2) in every district of each country either through a routine 2-dose vaccination schedule or through supplementary immunization activities (SIAs),§ 3) conduct high-quality, case-based surveillance in all countries, and 4) provide optimal clinical case management, including supplementing diets with vitamin A (1). Although significant progress was made toward measles elimination in the EMR during 1997–2007, the measles elimination goal was not reached by the target date of 2010, and the date was revised to 2015. This report updates previous reports (2–4) and summarizes the progress made toward measles elimination in EMR during 2008–2012. From 2008 to 2012, large outbreaks occurred in countries with a high incidence of measles,¶ and reported annual measles cases in EMR increased from 12,186 to 36,456. To achieve measles elimination in EMR, efforts are needed to increase 2-dose vaccination coverage, especially in countries with high incidence of measles and in conflict-affected countries, and to implement innovative strategies to reach populations at high risk in areas with poor access to vaccination services or with civil strife.

## Immunization Activities

Of the 23 EMR countries in 2012, administration of MCV1 was recommended at age 9 months in 12 (52%) countries and at age 12–15 months in 11 (48%) (Table 1). Twenty (87%) countries had measles vaccination schedules with at least 2 MCV doses. Reported vaccination coverage with MCV1 and MCV2 is calculated annually for each country by dividing the total number of doses administered to children in the targeted age group by the estimated population of children in that age group based on the most recent census (i.e., administrative coverage). Additionally, WHO and the United Nations Children’s Fund (UNICEF) estimated MCV1 coverage annually for each country using reported MCV1 coverage and available survey results (5). Estimated MCV1 coverage in EMR increased from 83% in 2008 to 85% in 2010 and then declined to 83% in 2012 (Table 1, Figure).

In 2012, estimated MCV1 coverage was unavailable for one of the 23 EMR countries, <90% (range = 46%–85%) in 10 (43%) countries, 90%–94% in two (9%) countries, and ≥95% in 10 (43%) countries (Table 1). Of the 10 countries with ≥95% MCV1 coverage, five reported ≥95% coverage in all districts. In 2012, among the 20 countries with a routine ≥2-dose schedule, reported MCV2 coverage was ≥95% in 11 (55%), 50%–94% in six (30%), and <50% in three (15%). During 2008–2012, a total of 186,760,207 doses of measles vaccine were given to children in 93 measles SIAs conducted in 15 EMR countries (Table 2). Of these SIAs, 38 (41%) had reported administrative coverage of ≥95%.

## Case-Based Surveillance Activities

Measles case-based surveillance includes individual case investigation and blood specimen collection for laboratory testing (6). Confirmation of measles is made by laboratory findings, an epidemiologic link,** or clinical diagnosis (6). By the end of 2012, nationwide measles case-based surveillance was established in all EMR countries except Somalia, South Sudan, and Pakistan, which had case-based surveillance at sentinel sites. Case-based surveillance was established nationwide in Djibouti; however, measles case information and surveillance performance indicators have not been reported from Djibouti to EMRO since February 2012.

An EMR Measles and Rubella Laboratory Network was established as part of the WHO Global Measles and Rubella Laboratory Network, with a national laboratory in each country and regional reference laboratories in Oman and Tunisia. National laboratories perform confirmatory testing of specimens from persons with suspected cases of measles using an enzyme-linked immunosorbent assay to detect measles-specific immunoglobulin M. In 2012, 18 (78%) of the 23 national laboratories also had capacity to perform measles virus isolation and polymerase chain reaction testing for viral detection. In 2012, 21 (91%) of the 23 national laboratories participated in and passed the laboratory proficiency panel testing and achieved accreditation by the Global Measles and Rubella Laboratory Network.

WHO global standards are used in EMR to monitor national case-based surveillance performance (7).†† In 2012, among 19 countries with reported performance indicators, 15 (79%) met the target of two or more discarded cases per 100,000 population, 15 (79%) met the target for adequacy of case investigation, 18 (95%) met the target for adequacy of specimen collection, and 14 (74%) met the target for adequacy of viral detection of outbreaks. Timeliness of transport to the laboratory and timeliness of laboratory reporting targets were achieved by 12 (63%) and 17 (89%) countries, respectively.

## Measles-Incidence and Measles Virus Genotypes

From 2008 to 2012, the annual number of EMR measles cases increased from 12,186 to 36,456, with an increase in measles incidence from 21.4 to 59.5 cases per million population. Large measles outbreaks occurred in countries with conflict and insecurity or with a high incidence of measles (Figure), including Djibouti (709 cases, 2012), Iraq (35,822 cases, 2008–2009), Pakistan (16,753 cases, 2010–2012), Somalia (27,281 cases, 2011–2012), South Sudan (3,208 cases, 2011–2012), Sudan (14,139 cases, 2011–2012), and Yemen (4,843 cases, 2011–2012). In addition, outbreaks with >1,500 measles cases were reported annually in Afghanistan during 2008–2012. In 2012 in EMR, >90% of measles cases occurred in those eight countries, which together had a measles incidence of 105.3, compared with 7.9 per million population in the other countries. In 2012, six countries with strong surveillance systems (i.e., Bahrain, Egypt, Oman, the West Bank and Gaza Strip, Syria, and Tunisia) reported a measles incidence of fewer than five cases per 1 million population.

During 2008–2012, genotype B3 was reported from 15 of 16 EMR countries that reported genotype results and was the predominant measles virus genotype detected. In contrast, genotype D4 was the predominant strain circulating during 2003–2007 (3).

### Discussion

Since EMR countries first resolved to eliminate measles, substantial progress has been made. During 2000–2012, measles incidence decreased 34%, from 90 to 59.5 per 1 million population, and estimated measles mortality decreased 52%, from 53,900 to 25,800 deaths per year (8). However, during 2008–2012, regional progress stagnated, and the number of reported measles cases increased more than two-fold, mainly because of large outbreaks in several countries. During 2008–2012, >80% of reported measles cases were from Afghanistan, Djibouti, Iraq, Pakistan, Somalia, South Sudan, Sudan, and Yemen. Increased civil conflict and insecurity in several countries since 2011 coincided with an increase in reported measles cases. With the resurgence of measles in some EMR countries, the region’s target of measles elimination by 2015 is not likely to be achieved.

Countries in the EMR face several challenges in achieving measles elimination. To achieve the “herd immunity” needed to interrupt endemic measles transmission, 2 doses of MCV with ≥95% coverage are needed. Routine MCV1 coverage remains suboptimal (83%) and, although 20 countries introduced MCV2 into the routine schedule, only half of these reported ≥95% MCV2 coverage. In addition, numerous SIAs were conducted; however, high coverage (≥95%) was not achieved in some countries. To prevent an accumulation of susceptible persons and subsequent measles outbreaks, a routine MCV2 dose should be introduced in all EMR countries and follow-up SIAs need to be conducted periodically until routine 2-dose coverage of ≥95% with both MCV1 and MCV2 is achieved and maintained in every district.

In certain countries where measles incidence remains high (notably Afghanistan, Pakistan, Somalia, South Sudan, Sudan, and Yemen), major challenges to implementing measles elimination activities exist, including civil unrest and armed conflict, competing public health priorities, and natural disasters. Unpredictable mass population displacements and resettlements complicate the delivery of routine vaccination services and planning of SIAs. Conducting SIAs in conflict settings and in areas with no local government requires establishing close linkages with local communities. Vaccination teams and civilian populations are at risk for violence during these SIAs, and vaccination coverage often is suboptimal.

During 2008–2012, measles case-based surveillance was implemented in all but three EMR countries, with the support of a well-established global and regional laboratory network. Measles case-based surveillance performance indicators showed that the majority of countries met surveillance standards. However, targets for surveillance indicators have not been met in all countries. Monitoring and strengthening surveillance performance could help rapidly identify and characterize outbreaks, guide response activities, and provide evidence for refining elimination strategies. Efforts also should be made to maintain sensitive, timely, and complete measles case-based surveillance in areas with conflict and insecurity.

What is already known on this topic?

Reported measles cases in the World Health Organization’s Eastern Mediterranean Region (EMR) decreased by 70%, from 146 per 1 million population in 1998 to 44 per 1 million in 2006. During 2000–2006, estimated measles deaths decreased by 73%. However, the goal of measles elimination by 2010 was not achieved, and the target date was revised to 2015.

What is added by this report?

During 2008–2012, estimated first-dose coverage with measles-containing vaccine in EMR was unchanged overall at 83%; approximately 200 million children were vaccinated during supplementary immunization activities (SIAs), and 38 (41%) of the 93 SIAs conducted had ≥95% national level administrative coverage. However, an increased number of measles cases were reported in 2012, a total of 36,456 compared with 12,196 in 2008. The increase was primarily caused by large measles outbreaks in countries with a high incidence of measles.

What are the implications for public health practice?

Successful implementation of all components of the EMR measles elimination strategy will be needed to achieve the regional goal of measles elimination by 2015. Efforts must be strengthened at the regional and national level to increase coverage with 2 doses of measles-containing vaccine, conduct high-quality SIAs, and use innovative strategies to reach high-risk populations living in areas with poor access or with civil strife.

The findings in this report are subject to at least two limitations. First, routine MCV1 and MCV2 administrative coverage and vaccination coverage during SIAs are likely to include errors resulting from inaccurate estimates of the size of the target population, inaccurate reporting of doses delivered, and inclusion of SIA doses given to children outside the target group. Second, underestimation in surveillance data can occur, because not all persons with suspected measles seek care and not all of those who seek care are reported.

To achieve measles elimination, the key strategies outlined in the Global Vaccine Action Plan and the Measles and Rubella Initiative Strategic Plan need to be implemented in all EMR countries (9,10). Efforts should focus on increasing MCV1 and MCV2 vaccination coverage and ensuring that routine immunization services and SIAs reach at-risk populations who reside in areas with poor access to vaccination services or with civil strife.

## Figures and Tables

**FIGURE:**
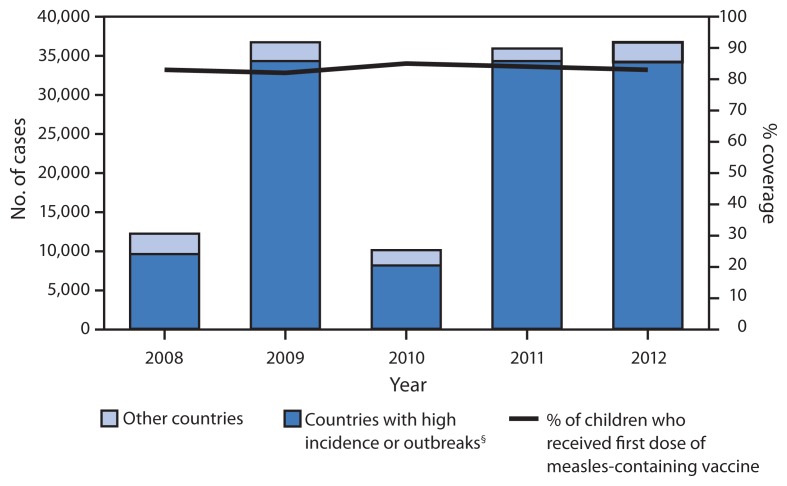
Number of reported measles cases,* by country’s measles status, and estimated percentage of children who received their first dose of measles-containing vaccine^†^ — World Health Organization (WHO) Eastern Mediterranean Region, 2008–2012 * Confirmed cases of measles reported to WHO and the United Nations Children’s Fund (UNICEF) through the Joint Reporting Form Regional Office for the Eastern Mediterranean Region. ^†^ By age 12 months or later if first dose was scheduled after age of 12 months. Data are from WHO and UNICEF estimates. ^§^ Countries with high incidence or outbreaks were Afghanistan, Djibouti, Iraq, Pakistan, Somalia, South Sudan, Sudan, and Yemen.

**TABLE 1:** Recommended 2012 national routine measles vaccination* schedule, estimated coverage with the first dose of measles-containing vaccine,† number of measles cases and measles cases, per 1 million population, by country — World Health Organization (WHO) Eastern Mediterranean Region, 2008 and 2012

				2008			2012		
					
Country/Area	Age at first dose	Age at second dose	Age at third dose	% coverage with MCV1§	No. of measles cases (JRF)§	Measles cases per 1 millon population	% coverage with MCV1§	No. of measles cases (JRF)§	Measles cases per 1 millon population
Afghanistan	9 mos¶	18 mos¶		59	1,599	59.2	68**	2,787	93.4
Bahrain	12 mos	5 yrs		99	2	1.8	99	0	0.0
Djibouti	9 mos¶	15 mos¶		73	143	176.5	83**	709	824.4
Egypt	12 mos	18 mos		92	668	8.8	93**	245	3.0
Iran	12 mos	18 mos		98	127	1.7	98	332	4.3
Iraq	9 mos¶	15 mos	4 yrs	76	5,494	186.7	69**	15	0.5
Jordan	9 mos¶	12 mos	18 mos	95	2	0.3	98	3	0.4
Kuwait	12 mos	2 yrs	12 yrs††	99	66	24.4	99	27	8.3
Lebanon	9 mos¶	12 mos	4–5 yrs	79	24	5.7	80**	9	1.9
Libya	12 mos	18 mos		98	8	1.4	98	320	52.0
Morocco	9 mos¶	None		96	1,455	47.0	99	668	20.5
Oman	12 mos	18 mos		98	18	6.9	99	13	3.9
Pakistan	9 mos¶	15 mos¶		81	1,129	6.8	83**	8,046	44.9
West Bank/Gaza Strip	12 mos	18 mos		96	0	0.0	N/A	0	0.0
Qatar	12 mos	18 mos		96	0	0.0	97	160	78.0
Saudi Arabia	9 mos¶	12 mos	6 yrs	97	158	5.4	98	294	10.4
Somalia	9 mos¶	None		34	1,081	118.3	46**	9,983	979.2
South Sudan	9 mos¶	None					62**	1,952	180.1
Sudan§§	9 mos¶	18 mos¶		79	129	3.8	85**	8,523	229.1
Syria	12 mos	18 mos		81	19	0.9	61**	13	0.6
Tunisia	15 mos¶	6 yrs¶	12 yrs††	98	2	0.2	96	48	4.4
UAE	12 mos	5–6 yrs		92	55	8.1	94**	132	14.3
Yemen	9 mos¶	18 mos¶		73	7	0.3	71**	2,177	91.3
**Region overall**				**83**	**12,186**	**21.4**	**83** **	**36,456**	**59.5**

**Abbreviations:** MCV1 = first dose of measles-containing vaccine; JRF = Joint Reporting Form; N/A = not available; UAE = United Arab Emirates.

* A combined measles, mumps, and rubella (MMR) vaccine is used except where noted.

† By age 12 months or later if first dose was scheduled after age 12 months. Data are from WHO and United Nations Children’s Fund (UNICEF) estimates.

§ Data available at http://www.who.int/immunization/monitoring_surveillance/data/subject/en.

¶ Single-antigen measles vaccine used, except in Tunisia, which uses monovalent measles vaccine at 15 months and measles-rubella vaccine at 6 years.

** Vaccination coverage was below the regional goal of ≥95% in 2012.

†† Third measles dose is given to girls at age 12 years (MMR vaccine in Kuwait and measles-rubella vaccine in Tunisia).

§§ Includes partial data for South Sudan.

**TABLE 2:** Measles supplementary immunization activities (SIAs),* by country/area, target age group, type of SIA, and number and percentage of targeted children vaccinated — World Health Organization (WHO) Eastern Mediterranean Region, 2008–2012

Country/Area	Year	Target age group	Type of SIA	Targeted children vaccinated	

				No.	(%)†
Afghanistan	2009	9–36 mos	Follow-up	3,000,777	(108)
	2011	9–59 mos	Mop-up	224,074	(98)
	2011	9–59 mos	Mop-up	200,470	(90)
	2011	9 mos–10 yrs	Mop-up	1,005,966	(96)
	2012	9 mos–10 yrs	Follow-up	6,194,612	(104)
	2012	9 mos–10 yrs	Follow-up	5,326,038	(103)
Djbouti	2008	9 mos–15 yrs	Catch-up	184,638	(86)
	2011	9–24 mos	Follow-up	4,866	(86)
	2012	9–59 mos	Follow-up	90,603	(95)
	2012	6–15 yrs	Catch-up	23,605	(94)
Egypt	2008	10–20 yrs	Catch-up	18,375,015	(99)
	2009	2–11 yrs	Catch-up	17,843,885	(104)
Iran	2010	9 mos–12 yrs	Mop-up	117,009	(99)
	2012	9 mos–12 yrs	Mop-up	142,730	(97)
Iraq	2008	7–36 mos	Mop-up	52,673	(108)
	2008	12–59 mos	Mop-up	198,075	(96)
	2008	9–59 mos	Mop-up	38,046	(70)
	2008	9–59 mos	Mop-up	154,369	(98)
	2009	6 yrs	Catch-up	1,070,243	(90)
	2009	9–60 mos	Follow-up	180,699	(99)
	2009	6–59 mos	Follow-up	4,513,438	(96)
	2009	5–12 yrs	Follow-up	5,380,608	(88)
	2010	9–59 mos	Follow-up	2,603,752	(93)
	2010	9 mos–12 yrs	Mop-up	117,009	(99)
	2011	18–24 yrs	Catch-up	1,849,139	(40)
	2012	6 mos–5 yrs	Follow-up	4,733,889	(94)
Jordan	2012	9–59 mos	Mop-up	163,001	(90)
Kuwait	2010	1–7 yrs	Follow-up	272,829	(75)
Lebanon	2008	9 mos–15 yrs	Catch-up	705,117	(77)
Libya	2008	1–15 yrs	Mop-up	36,480	(100)
	2008	1–6 yrs	Mop-up	1,550	(100)
	2009	12 mos–6 yrs	Follow-up	748,345	(98)
Morocco	2008	9 mos–14 yrs	Catch-up	4,665,375	(99)
Pakistan	2008	9 mos–13 yrs	Catch-up	35,315,375	(103)
	2010	9 mos–13 yrs	Mop-up	4,159,306	(81)
	2010	9 mos–13 yrs	Mop-up	1,583,340	(93)
	2010	6 mos–59 mos	Mop-up	7,998,260	(96)
	2011	6–59 mos	Follow-up	1,229,618	(93)
	2011	6–59 mos	Follow-up	5,098,071	(99)
	2011	6–59 mos	Follow-up	784,337	(90)
	2011	9–59 mos	Follow-up	1,744,206	(86)
	2011	9–59 mos	Follow-up	205,551	(91)
	2011	9–59 mos	Follow-up	547,716	(98)
	2012	9 mos–9 yrs	Follow-up	1,954,175	(102)
Qatar	2011	12 mos–20 yrs	Follow-up	150,112	(77)
Saudi Arabia	2011	6–18 yrs	Catch-up	4,900,677	(97)
	2011	9 mos–6 yrs	Catch-up	3,369,639	(97)
Somalia	2008	9 mos–15 yrs	Mop-up	142,654	(95)
	2008	9 mos–15 yrs	Mop-up	138,205	(58)
	2009	9–59 mos	Follow-up	119,117	(82)
	2009	9–59 mos	Follow-up	325,622	(90)
	2009	9–59 mos	Follow-up	214,864	(87)
	2009	9–59 mos	Follow-up	276,994	(73)
	2009	9–59 mos	Follow-up	137,699	(95)
	2009	9–59 mos	Follow-up	835,927	(82)
	2009	9–59 mos	Follow-up	909,687	(85)
	2010	9–59 mos	Follow-up	291,966	(86)
	2010	9–59 mos	Follow-up	327,591	(86)
	2010	9–59 mos	Follow-up	1,137,268	(92)
	2011	9–59 mos	Mop-up	75,197	(89)
	2011	6 mos–15 yrs	Mop-up	71,653	(80)
	2011	9–59 mos	Follow-up	151,279	(89)
	2011	9–59 mos	Follow-up	323,986	(85)
	2011	6 mos–14 yrs	Mop-up	1,056,287	(36)
	2011	6 mos–15 yrs	Mop-up	656,226	(88)
	2011	6 mos–15 yrs	Mop-up	74,300	(86)
	2011	6 mos–14 yrs	Mop-Up	626,625	(93)
	2012	6–59 mos	Follow-up	509,042	(87)
	2012	<5 yrs	Follow-up	886,033	(87)
	2012	9–59 mos	Follow-up	872,230	(91)
Sudan	2008	9–59 mos	Follow-up	2,728,011	(97)
	2008	6 mos–14 yrs	Catch-up	150,619	(83)
	2008	9 mos–5 yrs	Follow-up	142,511	(94)
	2010	9–59 mos	Mop-up	313,359	(97)
	2010	9–59 mos	Follow-up	1,763,398	(95)
	2011	9 mos–15 yrs	Mop-up	64,063	(67)
	2011	9–59 mos	Follow-up	1,020,921	(105)
	2011	9–59 mos	Follow-up	1,456,371	(102)
	2011	9–59 mos	Follow-up	1,433,328	(92)
	2011	9–59 mos	Mop-up	68,994	(78)
South Sudan	2008	6 mos–14 yrs	Catch-up	132,282	(66)
	2011	6–59 mos	Follow-up	678,503	(102)
	2011	6–59 mos	Follow-up	502,258	(92)
	2011	6–59 mos	Follow-up	146,644	(99)
	2011	6 mos–14 yrs	Follow-up	186,459	(93)
	2012	6–59 mos	Follow-up	1,708,418	(90)
Syria	2008	11–15 yrs	Catch-up	1,610,305	(100)
	2012	12–59 mos	Follow-up	768,086	(60)
Yemen	2009	9–59 mos	Mop-up	621,671	(93)
	2009	9–59 mos	Follow-up	3,246,804	(96)
	2010	6 mos–15 yrs	Mop-up	455,517	(76)
	2011	6 mos–15 yrs	Mop-up	26,241	(85)
	2011	6–59 mos	Mop-up	130,905	(65)
	2012	6 mos–10 yrs	Follow-up	7,984,779	(93)
**Region overall**				**186,760,207**	

* SIAs generally are carried out using two approaches. An initial nationwide catch-up SIA targets all children aged 9 months to 14 years; it has the goal of eliminating susceptibility to measles in the general population. Periodic follow-up SIAs then target all children born since the last SIA. Follow-up SIAs generally are conducted nationwide every 2–4 years, targeting children aged 9–59 months; their goals are to eliminate measles susceptibility that has developled in recent birth cohort and to protect chidren who did not respond to the first measles vaccination. The exact age range for follow-up SIAs depends on the age-specific incidence of measles, coverage with 1 dose of measles-containing vaccine, and the time since the last SIA.

† The percentage of the population vaccinated can exceed 100% because of underestimation of the size of the target population or data quality issues.

## Footnotes

* The 22 EMR countries were Afghanistan, Bahrain, Djibouti, Egypt, Iran, Iraq, Jordan, Kuwait, Lebanon, Libya, Morocco, Oman, Pakistan, Qatar, Saudi Arabia, Somalia, Sudan, Syria, Tunisia, United Arab Emirates, West Bank and Gaza Strip, and Yemen. For this report, the geographic regions West Bank and Gaza Strip are considered to constitute one country. In July 2011, South Sudan became an independent nation, for a total of 23 states in the region.

† Measles elimination is defined as the absence of endemic measles cases for a period of ≥12 months, in the presence of adequate surveillance. One indicator of measles elimination is a sustained measles incidence of less than one case per 1 million population.

§ Initial nationwide catch-up SIAs in EMR countries target all children aged 9 months–14 years, with the goal of eliminating susceptibility to measles in the general population. Periodic follow-up SIAs target all children born since the last SIA. Follow-up SIAs generally are conducted nationwide every 2–4 years and target children aged 9–59 months, with the goals of eliminating any measles susceptibility that has developed in recent birth cohorts and protecting children who did not respond to their first measles vaccination.

¶ Countries with high incidence of measles were Afghanistan, Djibouti, Pakistan, Somalia, South Sudan, Sudan, and Yemen.

** An epidemiologic link is defined as a clinical case of measles that has not been confirmed by a laboratory but that is geographically and temporally related (with dates of rash onset occurring 7–21 days apart) to a laboratory-confirmed case.

†† These indicators include ensuring that 1) two or more nonmeasles suspected cases per 100,000 persons per year are detected and reported (to monitor the sensitivity of the surveillance system), 2) ≥80% of suspected measles cases have an adequate investigation initiated within 48 hours of notification with essential data elements collected (to monitor adequacy of investigation), 3) ≥80% of suspected cases have adequate specimens collected and tested in a proficient laboratory (to monitor adequacy of testing), 4) ≥80% of confirmed outbreaks have adequate specimens collected for viral detection in a proficient laboratory (to monitor virus transmission), 5) ≥80% of specimens are received by a laboratory within 5 days of collection (to monitor timely specimen transport), and 6) ≥80% of laboratory test results are reported within 4 days (to monitor timely testing and reporting).
